# Plasticity in induced resistance to sequential attack by multiple herbivores in *Brassica nigra*

**DOI:** 10.1007/s00442-021-05043-1

**Published:** 2021-10-13

**Authors:** Maite Fernández de Bobadilla, Roel Van Wiechen, Gerrit Gort, Erik H. Poelman

**Affiliations:** 1 Laboratory of Entomology, Wageningen University and Research Center, Droevendaalsesteeg 1, 6708 PB Wageningen, The Netherlands; 2 Biometris, Wageningen University and Research Center, Droevendaalsesteeg 1, 6708 PB Wageningen, The Netherlands

**Keywords:** Sequential herbivory, Multi-herbivore attack, Canalisation, Switching, Defence plasticity, *Plutella xylostella*

## Abstract

In nature, plants interact with multiple insect herbivores that may arrive simultaneously or sequentially. There is extensive knowledge on how plants defend themselves against single or dual attack. However, we lack information on how plants defend against the attack of multiple herbivores that arrive sequentially. In this study, we investigated whether *Brassica nigra* L. plants are able to defend themselves against caterpillars of the late-arriving herbivore *Plutella xylostella* L., when plants had been previously exposed to sequential attack by four other herbivores (*P. xylostella, Athalia rosae, Myzus persicae* and *Brevicoryne brassicae*). We manipulated the order of arrival and the history of attack by four herbivores to investigate which patterns in sequential herbivory determine resistance against the fifth attacker. We recorded that history of sequential herbivore attack differentially affected the capability of *B. nigra* plants to defend themselves against caterpillars of *P. xylostella.* Caterpillars gained less weight on plants attacked by a sequence of four episodes of attack by *P. xylostella* compared to performance on plants that were not previously damaged by herbivores. The number of times the plant was attacked by herbivores of the same feeding guild, the identity of the first attacker, the identity and the guild of the last attacker as well as the order of attackers within the sequence of multiple herbivores influenced the growth of the subsequent herbivory. In conclusion, this study shows that history of sequential attack is an important factor determining plant resistance to herbivores.

## Introduction

In nature, plants interact with a species-rich community of insects (Giron et al. 2018). Some interactions with insects are beneficial for the plant, such as those with pollinators, whereas others are detrimental such as interactions with herbivores. Plants are exposed to the attack of multiple insect herbivores that feed from their tissues simultaneously or sequentially. Because insect herbivores often have a negative effect on plant fitness, plants have evolved defence mechanisms to cope with insect herbivory (Erb 2018). Some of those defence mechanisms are constitutive, and are always expressed independently of the presence of the attacker (War et al. 2012). However, maintaining defences is metabolically costly, and herbivores are very diverse in the way they consume the plant and in the defence traits they are sensitive to. Therefore, plants have evolved induced defences that are only expressed upon attack and are more specific to the attacker compared to constitutive defence (Karban 2020).

Studies on plants under attack by two herbivore species identified that plant resistance to the second herbivore may be affected by induced responses to initial herbivore attack (Stam et al. 2014; Karban 2020). A response to initial attack may enhance resistance to a second herbivore when this herbivore is affected by similar plant traits or the response to the first herbivore includes priming for resistance to a second herbivore (Mertens et al. 2021a). However, herbivore-induced plant responses may also lead to susceptibility to attack by a second herbivore (Soler et al. 2012; Thaler et al. 2012; Ali et al. 2014; Moreira et al. 2018﻿). The induced susceptibility may arise when a response to the first herbivore depletes the resources available for an effective response to the second attacker (Herms and Mattson 1992; Züst and Agrawal 2017) or when the physiological response to the first attacker limits a response to the second attacker (Pieterse et al. 2012; Thaler et al. 2012). Cross-talk between plant signal transduction pathways to insect feeding has been identified to play an important role in fine tuning plant resistance to dual herbivore attack, but may also lead to the phenomenon of induced susceptibility (Pieterse et al. 2012; Soler et al. 2012; Thaler et al. 2012). When plants are sequentially attacked by two insects of a different guild, resistance may be impaired because the signal transduction pathway involved in responses to phloem-feeding herbivores may antagonistically interact with signal transduction to leaf-chewing herbivores (Erb et al. 2012; Koornneef and Pieterse 2008; Pieterse et al. 2012). However, a recent study on *Brassica nigra* identifies that not all interactions between phloem feeders and leaf chewers result in induced susceptibility, but that plants may have adapted their induced response to the first attacker depending on the likelihood of subsequent attack determined by the prevalence of a second attacker in the field (Mertens et al. 2021a, b).

Remarkably, only few studies have evaluated plant resistance beyond dual attack, even though plants are commonly in situations of multi-herbivore attack. These studies show that the order, the identity and the species richness of the attackers influence plant resistance against a late-arriving herbivore (Fernández de Bobadilla et al. 2021; Mathur et al. 2013; Stam et al. 2014, 2017, 2018, 2019). Nonetheless, in nature, herbivores often arrive sequentially, and studies including a realistic number of sequential attackers are lacking. This largely limits our understanding of how plastic plants adapt their resistance phenotype to attackers that arrive in sequences (Mertens et al. 2021a). Several factors may determine the plant’s ability to deal with sequential herbivore attack, such as the frequency of exposure to an attacker of a different feeding guild or the order of arrival of the attackers in the sequence such as the guild/identity of the first or last attacker in the sequence (Erb et al. 2011). Plants need to maximize their defence response to a current attacker in the context of an optimal response to the dynamic community of herbivores that may arrive later and also affect plant fitness (Mertens et al. 2021a, b; Poelman and Kessler 2016; Orrock et al. 2015).

The aim of this study was to investigate whether plants are able to defend themselves against a late-arriving leaf-chewing herbivore, when they had been previously exposed to sequential attack by four other herbivores. We subjected black mustard (*B. nigra*) plants to attack by 12 different sequences of herbivory or left the plants without herbivory and investigated the performance of *Plutella xylostella* on the induced plants. Under natural conditions, individual *B. nigra* plants are attacked by at least four up to twelve different herbivore species over their life-time (Mertens et al. 2021b; Poelman et al. 2009). It is a common annual in Europe, invasive on other parts of the globe and is used by several research groups as a model plant for ecological and physiological studies of plant–insect interactions (van Dam et al. 2005; Lankau and Strauss 2008; Mertens et al. 2021b; Oduor et al. 2011; Papazian et al. 2019; Pashalidou et al. 2020). We manipulated the number of times there were leaf-chewing or phloem-feeding insects in the four incidences of herbivore attack, as well as the order of the attackers and their species identity. We hypothesized that plants attacked more frequently by a leaf chewer would be better prepared to respond to a late-arriving leaf chewer. Because the response to a first herbivore may most profoundly determine the capabilities of plants to deal with subsequent attack (Viswanathan et al. 2007), plants first attacked by a chewer may be better able to respond to a late-arriving chewer compared to plants first attacked by a phloem-feeding herbivore. Similarly, plants more recently attacked by a chewer may show a stronger induced resistance towards chewers, compared with plants more recently attacked by a phloem feeder. Thus, we hypothesized that when the most recent attacker (i.e. the last in a sequence of four) was a chewer, plants would be more resistant to a late-arriving chewer (fifth herbivore) than plants that had a phloem feeder as most recent attacker. Finally, we hypothesized that the order of the herbivores in the sequence would be an important factor in determining plant resistance against a late-arriving herbivore.

## Materials and methods

### Plants and insects

Two-and-a-half-week-old black mustard plants (*B. nigra*, Brassicales: Brassicaceae) were used for the experiments. We designed the experiment as such that the plants remained in vegetative stage during the entire experiment, to avoid that plants would alter physiology due to entering the bud or flowering stage and plants could sustain the sequence of herbivory not losing more than 50% of their leaf tissue. Seeds were obtained from a natural population in the vicinity of Wageningen (51° 57′ 32″ N, 5° 40′ 23″ E). The plants and the insects were grown and maintained in a greenhouse at 22 ± 2 °C, 60–70% RH and 16:8 h L:D photo regime. We used four herbivore species to simulate sequential attack: second instar larvae of the diamondback moth *P. xylostella* (*Px*) (Lepidoptera: Plutellidae) and first instar larvae the turnip sawfly, *Athalia rosae* (*Ar*) (Hymenoptera: Tenthredinidae) as leaf chewers and the cabbage aphid, *Brevicoryne brassicae* (*Bb*) and the green peach aphid, *Myzus persicae* (*Mp*) (both Hemiptera: Aphididae) as phloem feeders (Table 1). The degree of resistance induced by different sequences of attack by the four herbivores was determined by assessing the performance of second instars of the diamondback moth *P. xylostella* (Lepidoptera: Plutellidae) as fifth herbivore (that we call receiver). Larvae of this insect are specialists on brassicaceous plants and feed on foliar tissue, buds and flowers. *P. xylostella* typically arrives later in the vegetative growing season of *B. nigra* plants and often has to deal with plants previously damaged by other herbivores (Mertens et al. 2021b). *B. brassicae* and *P. xylostella* were reared on Brussels sprouts (*Brassica oleracea* L. var. *gemmifera* cv. Cyrus). *M. persicae* and *A. rosae* were reared on radish (*Raphanus sativus*). All insects were obtained from the stock rearing of the Laboratory of Entomology, Wageningen University.

**Table 1:**
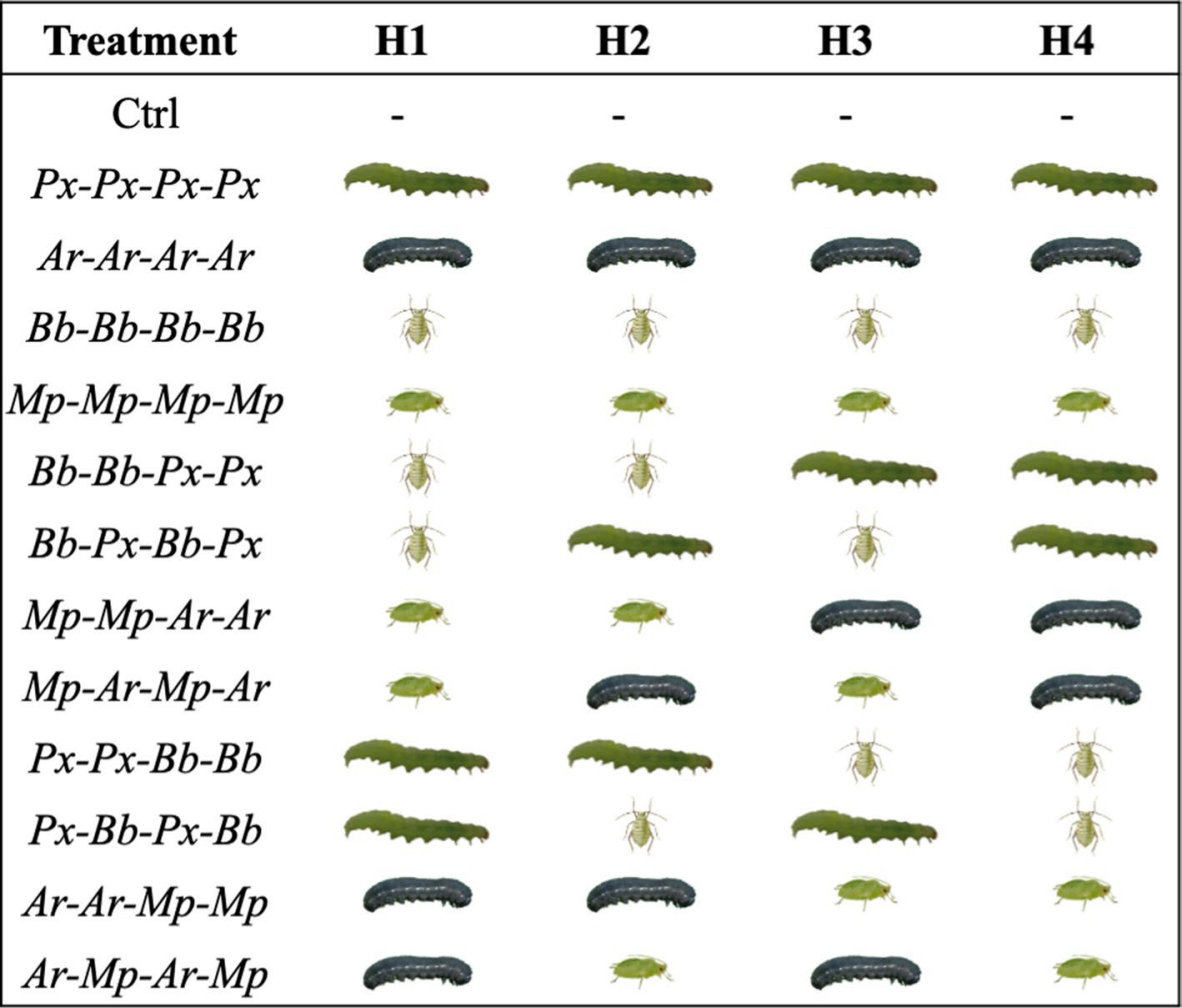
List of herbivore sequences (H) applied to *Brassica nigra* plants to assess plant resistance to *Plutella xylostella*

Leaf chewers: *Px* *P. xylostella*, *Ar* *Athalia rosae*. Phloem feeders: *Bb* *Brevicoryne brassicae*, *Mp* *Myzus persicae*

### Assessing herbivore performance on plants induced by a history of sequential attack by four herbivores

**Fig. 1:**
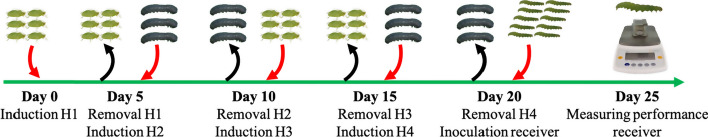
Timeline depicting the experimental design of sequential attack of four herbivores and their effect on performance of a fifth herbivore as measure of plant resistance to multi-herbivore attack. On day 0, each *Brassica nigra* plant was induced with the first set of herbivores (H1) which consisted of either three chewers or six aphids. After five days, all the herbivores were removed from the plant using a brush, and the second set was introduced (H2). This was done for four events of an herbivore sequence. After the herbivores of the last round of herbivory (H4) had been removed from the plant, 10 larvae of *Plutella xylostella* were introduced on each plant and they were allowed to feed from the induced plants for five days. Each *P. xylostella* larva was recaptured and weighed as a measure as of plant resistance

To test plant plasticity to multiple herbivore attack, we challenged *B. nigra* plants with several herbivore sequences in a greenhouse (19 °C, 60–70% RH and 16:8 h L:D photo regime). We assessed whether plant responses to the herbivore sequences differentially affected performance of caterpillars of *P. xylostella* as proxy for plant resistance (Fig. 1). We prepared a total of 18 plant replicates per herbivore combination divided in two blocks separated in time (nine plant replicates per herbivore combination in each block). We assessed induced plant responses to sequential attack by four herbivores, with a total of 12 treatments that differed in the order and identity of herbivore attack (Table 1). We used a full factorial design in which we specifically manipulated the number of times plants were attacked by leaf-chewing or phloem-feeding insects, as well as the order of the attackers and their species identity. For each of the four episodes of herbivory in a sequence, the total number used was three leaf chewers or six phloem feeders. For the chewers, first instar larvae for *A. rosae* and second instar larvae for *P. xylostella* (as the first instar of these larvae is a leaf miner) were used. For the phloem feeders, middle-sized adults were used, allowing for population growth during the induction period. The number of individuals per species initially introduced is representative for natural herbivore communities on *B. nigra* plants (Mertens et al. 2021b). We also prepared control plants that did not receive any inducing herbivore, but were otherwise treated in a similar way as plants receiving herbivores (i.e., on those plants only caterpillars of the receiving herbivore *P. xylostella* were introduced to measure the baseline of performance of this species on undamaged plants). Each herbivore episode in the sequence lasted for a period of five days. Herbivores were placed on the youngest fully expanded leaf and were allowed to freely move and feed from the plant. They were prevented from moving to neighbouring plants by placing plants in inundated trays and by spacing out plants with at least 40 cm between plants. After these five days, all the inducers were removed with a fine brush to exclude direct effects of inducing herbivores on the next herbivore in the sequence and on the receiving herbivore. By removing herbivores, we could focus on distinct events of herbivory, without aphid populations or chewing herbivores overexploiting plants due to the absence of control by their natural enemies. After plants had been exposed to their treatment of sequential attack by a sequence of four herbivore species and these herbivores had been removed, each plant was infested with 10 s instar larvae of *P. xylostella*, which acted as receiver to assess whether their performance was differentially affected by the history of herbivore attack (Fig. 1). The growth of *P. xylostella* larvae was measured after five days of feeding on the induced plants as a proxy of plant resistance. After five days of feeding, the caterpillars are in or close to their final instar and fastest growing caterpillars are not yet pupating. The five-day performance covers about 80% of the larval development time of this herbivore species. The starting mass of the caterpillars was not measured since all caterpillars were from the same age and their mass was below the analytical error of the balance. Weight of each individual caterpillar after five days of feeding was assessed on a Sartorius^®^—CP2P—Analytical Balance (accuracy 0.001 mg).

### Statistical analysis

To investigate whether the history of herbivory on *B. nigra* affected the growth of larvae of *P. xylostella*, we performed several analyses. For all tests, we fitted a Mixed Linear Model (MLM), using as fixed effects the time blocks (with two levels) and appropriate part of the data grouped to answer the question of interest. Plants were included as random factors and residual error was also included. First, to assess whether the herbivore sequences influenced the performance of larvae of *P. xylostella* feeding as fifth herbivore, we used fixed effects for all the treatments (with 13 levels: 12 herbivore sequences and a control). Second, to compare the treatments that received four times the same herbivores, we used fixed effects for the selected treatments (with five levels: control, and four sequences of either *P. xylostella, A. rosae, B. brassicae* or *M. persicae*). Third, to explore whether the number of times the plant was attacked by an insect of the same feeding guild affected performance of *P. xylostella*, we grouped our data based on the number of switches of chewers or of aphids in the four episodes of sequential herbivory, using this time fixed effects for number of sequences of chewers/aphids (with four levels: control, zero, two or four). For the rest of the analyses, we excluded the data from the treatments that received four times the same herbivore. We did so because we were interested in comparing the effect of herbivore sequences that contained an equal number of switches of attackers in the sequence. First, we used fixed effects for guild of the first or of the last attacker (with three levels: control, chewer or aphid). Then, we used fixed effects for species identity (with five levels: control, *P. xylostella, A. rosae, B. brassicae* and *M. persicae*). Finally, we tested for the effect of order of herbivores within the sequence, and compared treatments that received the same herbivores but in different order.

## Results

### History of sequential herbivore attack affects performance of larvae of *P. xylostella*

**Fig. 2:**
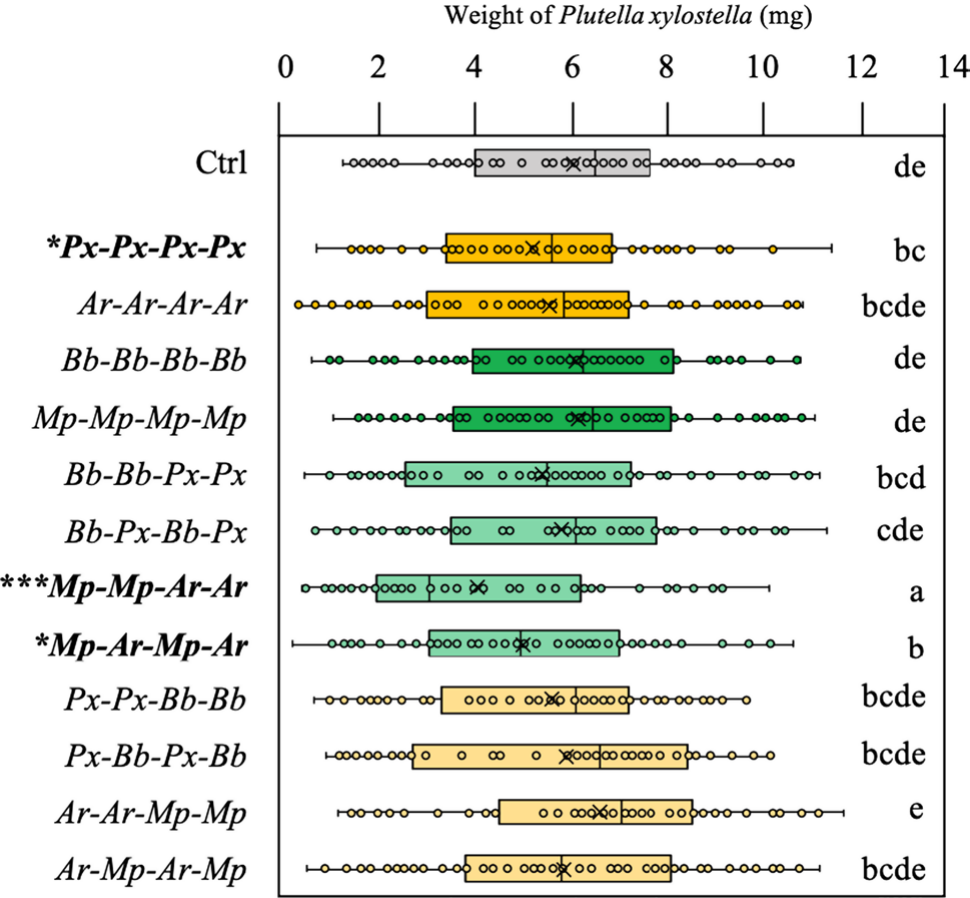
Specificity in the effect of attacker sequence of multi-herbivore attack on resistance to a late-arriving herbivore. Weight (mg) of *Plutella xylostella* larvae after feeding for five days from *Brassica nigra* plants previously attacked by sequences of four herbivore events (*N* = 18). Dark yellow and dark green boxplots indicate herbivore sequences that received four times a chewer or a phloem feeder, respectively. Light yellow and light green boxplots indicate herbivore sequences of two chewers plus two phloem feeders, where the first attacker was a chewer or a phloem feeder, respectively. Herbivore sequences that affected *P. xylostella* growth (compared to control, untreated plants) are marked in bold and with asterisks with significance levels *P < 0.05; ***P < 0.001. Boxplot height corresponds to the first and third quartiles (Q1 and Q3), and the middle line to the median. Letters above the boxplots show significant differences (MLM, posthoc Tukey). Leaf chewers: *Plutella xylostella* (*Px*) and *Athalia rosae* (*Ar*). Phloem-feeding aphids *Brevicoryne brassicae* (*Bb*) and *Myzus persicae* (*Mp*)

The sequence of herbivore attack affected the capability of *B. nigra* plants to defend against larvae of *P. xylostella* (*F*_1,12_ = 3.42, *P* < 0.001, Fig. 2). A sequence of attack by four episodes of leaf-chewing caterpillars of *P. xylostella* reduced the growth of larvae of *P. xylostella* that were feeding on these plants as fifth herbivore (*t*_1,2_ =  − 2.17, *P* = 0.032, Fig. 2). No effect on insect growth was induced by a sequence of four episodes of attack by the other leaf chewer *A. rosae.* Performance of *P. xylostella* caterpillars feeding on plants induced by a history of four rounds of *A. rosae* did not differ from *P. xylostella* caterpillars feeding on plants that did not receive herbivory. Sequential attack by four rounds of aphid attack by either *M. persicae* or *B. brassicae* did not affect the performance of *P. xylostella* caterpillars as compared to performance on undamaged plants. However, specific sequences of herbivore attack affected the performance of *P. xylostella* caterpillars compared to performance on undamaged plants. *P. xylostella* larvae grew less on plants that had been exposed to sequential attack by *M. persicae*–*M. persicae*–*A. rosae*–*A. rosae* (*t*_1,2_ =  − 4.42, *P* < 0.001, Fig. 2) and by *M. persicae*–*A. rosae*–*M. persicae*–*A. rosae* (*t*_1,2_ = -2.34, *P* = 0.020, Fig. 2).

### Importance of specific events in sequential attack for performance of *P. xylostella* caterpillars

The differential effect of specific orders of sequential herbivore attack on performance of *P. xylostella* was determined by the number of switches between herbivore guilds, the identity of the first and last herbivore as well as the specific order of herbivore attack.

**Fig. 3:**
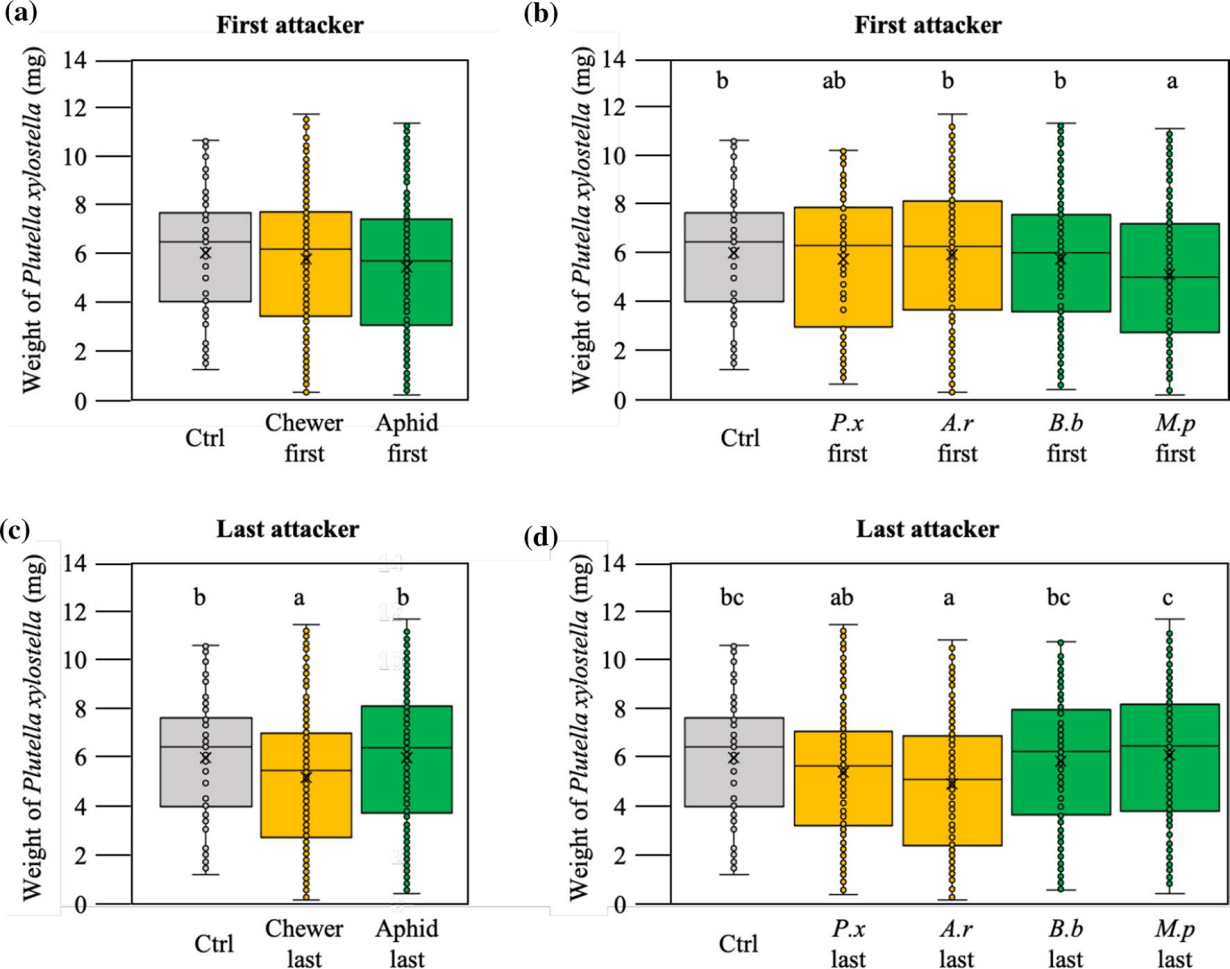
Both the first and last herbivore in a sequence of four affects plant resistance to the fifth attacker *Plutella xylostella*. Weight (mg) of *P. xylostella* larvae after feeding as fifth herbivore from *Brassica nigra* plants that were previously attacked by four sets of herbivores where: **a** the first attacker was either a chewer, an aphid or untreated plants (Ctrl). **b** The first attacker was *Plutella xylostella* (*Px*), *Athalia rosae* (*Ar*), *Brevicoryne brassicae* (*Bb*), *Myzus persicae* (*Mp*), or untreated plants (Ctrl). **c** The last attacker was either a chewer, an aphid or untreated plants (Ctrl). **d** The last attacker was *Plutella xylostella* (*Px*), *Athalia rosae* (*Ar*), *Brevicoryne brassicae* (*Bb*), *Myzus persicae* (*Mp*), or untreated plants (Ctrl). Boxplot height corresponds to the first and third quartiles (Q1 and Q3), and the middle line to the median. Letters above the boxplots show significant differences (MLM, posthoc Tukey)

First, the number of times a plant was exposed to the same feeding guild of attacker as part of sequential herbivore attack affected the growth of larvae of *P. xylostella* (*F*_1_,_3_ = 3, *P* = 0.04). Larvae of *P. xylostella* grew less on plants that were attacked four times by chewers than on plants exposed to four times attack by phloem feeders (*t*_1,2_ =  − 1.67, *P* = 0.097) that had similar performance to *P. xylostella* feeding from control plants. Twice an attack by the same feeding guild on average did not differ from control plants. Second, the feeding guild of the first attacker of the sequence did not affect the growth of larvae of *P. xylostella* (Fig. 3a). However, the specific species identity of the first attacker of the sequence affected performance of *P. xylostella* (MLM: *F*_1_,_4_ = 2.89, *P* = 0.023, Fig. 3b). Plants that had been attacked first by *M. persicae* sustained reduced performance of *P. xylostella* independent of the order and identity of the second, third and fourth herbivore in the sequence (*t*_1,2_ =  − 2.33, *P* = 0.02, Fig. 3b). Third, the feeding guild as well as the species identity of the last and thus fourth attacker of the sequence affected performance of *P. xylostella* larvae (MLM, Guild: *F*_1_,_3_ =  − 8.12, *P* < 0.001; Identity: *F*_1_,_4_ = 5.11, *P* < 0.0001, Fig. 3c, d). *P. xylostella* larvae grew less when the last attacker had been a chewer (MLM, chewer *t*_1,2_ =  − 2.33, *P* = 0.021) and these effects were particularly apparent for the leaf chewer *A. rosae* (MLM, *t*_1,4_ =  − 2.78, *P* = 0.006, Fig. 3c, d).

**Fig. 4:**
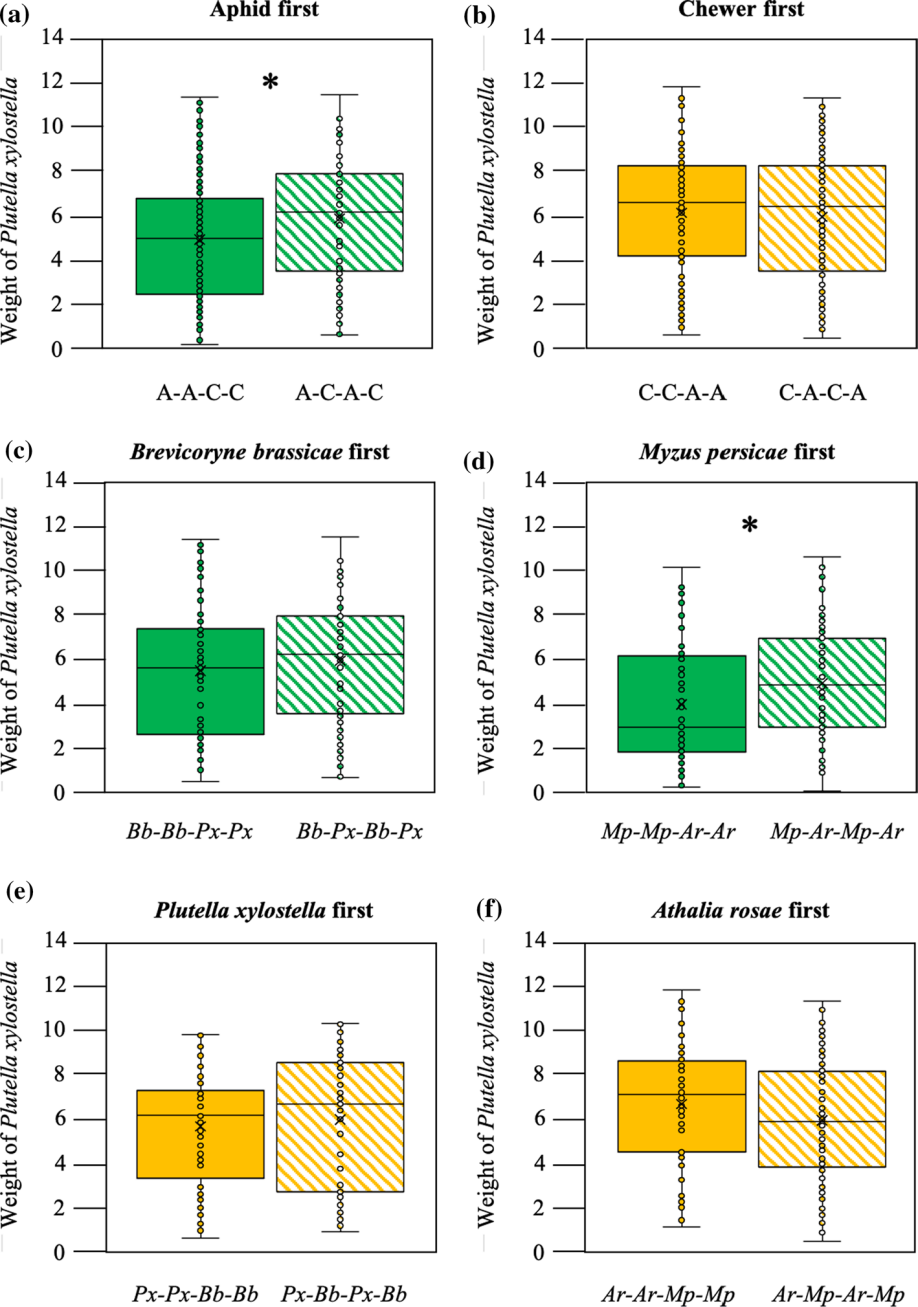
Feeding guild and species identity of first herbivore in sequence of multi-herbivore attack differentially affects performance of the fifth herbivore. Weight (mg) of *Plutella xylostella* larvae after feeding for five days from *Brassica nigra* plants attacked by a sequence of herbivory with the guild or the species identity of first attacker constant but changing the order of the subsequent attackers of the sequence. First attacker: **a** an aphid (A), **b** a chewer (C), **c** *Brevicoryne brassicae* (*Bb*) **d** *Myzus persicae,* (*Mp*) **e** *Plutella xylostella* (*Px*), **d** *Athalia rosae* (*Ar*). Boxplot height corresponds to the first and third quartiles (Q1 and Q3), and the middle line to the median. Asterisks show comparisons with significance differences *P < 0.05

To further separate the effects caused by number of switches between feeding guilds from effects by feeding guild, species identity and order of arrival, we analysed these effects within subsets of treatments that were equal in the number of herbivore switches. *P. xylostella* larvae gained more weight on *B. nigra* plants attacked by sequences containing two times chewers, and two times phloem feeders, when the first attacker was a chewer, compared with plants that were first attacked by a phloem feeder (*F*_1,2_ = 10.85, *P* = 0.001). After first attack by a phloem feeder, the order of subsequent attackers influenced performance of *P. xylostella*, as larvae grew larger on plants attacked by the sequence aphid–chewer–aphid–chewer (*B. brassicae*–*P. xylostella*–*B. brassicae*–*P. xylostella* as well as *M. persicae*–*A. rosae*–*M. persicae*–*A. rosae*) than on plants attacked by the sequence aphid–aphid–chewer–chewer (*B. brassicae*–*B. brassicae*–*P. xylostella*–*P. xylostella* as well as *M. persicae*–*M. persicae*–*A. rosae*–*A. rosae*) (MLM, *F*_1,2_ = 5.06, P = 0.03; Fig. 4a). When the first attacker was the phloem feeder *B. brassicae,* the performance of *P. xylostella* was similar*,* irrespectively of the order of the subsequent attackers (*F*_1,2_ = 1.34 P = 0.25, Fig. 4c). In contrast, when the first attacker was *M. persicae*, the order of the attackers mattered and larvae of *P. xylostella* grew more on plants where the identity of the herbivore switched every time (larvae grew more on plants that had been exposed to *M. persicae*–*A. rosae*–*M. persicae*–*A. rosae* than on plants that had been exposed to *M. persicae*–*M. persicae*–*A. rosae*–*A. rosae*) (*F*_1,2_ = 4.08 *P* = 0.04, Fig. 4d). In contrast to the effect of herbivore order when the first attacker was an aphid, the order of herbivore arrival after the first herbivore was a leaf chewer did not affect herbivore performance (*F*_1,2_ = 0.75 *P* = 0.39, Fig. 4b). These effects were also similar for the two leaf chewers *P. xylostella* or *A. rosae* (*P. xylostella* first: *F*_1,2_ = 0.05 *P* = 0.83; *A. rosae* first: *F*_1,2_ = 2.08 *P* = 0.15, Fig. 4e, f).

## Discussion

The objective of this study was to investigate plant resistance against a leaf-chewing herbivore, after plants had been previously exposed to sequential attack by four other herbivores. We found that sequence of herbivore attack differentially affected the performance of *P. xylostella* larvae on *B. nigra* plants Four events of attack by *P. xylostella* reduced the performance of larvae of *P. xylostella* compared to control plants. The number of times the plant was attacked by herbivores of the same feeding guild, the identity of the first attacker, the identity and the guild of the last attacker as well as the order of attackers within the sequence of multiple herbivory influenced the growth of *P. xylostella* larvae feeding from herbivore-induced plants. The guild of the first attacker of the sequence did not affect the performance of *P. xylostella*. However, when plants had been first attacked by *M. persicae* they grew less*,* regardless of the order of other herbivores attacking the plant. In contrast, the guild and identity of the last attacker influenced the growth of *P. xylostella* larvae as they grew less on plants where the last attacker of the sequence had been a chewer, especially when it was *A. rosae.* Our study shows that the sequence of herbivore attack is an important factor determining herbivore performance. Here, we discuss our findings in the context of plant defence plasticity to attack by multiple herbivores.

In line with the induced defence hypothesis, *B. nigra* plants that had been attacked four times by caterpillars of *P. xylostella* were more resistant to caterpillars of *P. xylostella* feeding as fifth herbivore as indicated by reduced caterpillar performance on induced plants compared to performance on undamaged plants. However, when the aphid *B. brassicae* was introduced in the sequence of four attackers the induced resistance to larvae of *P. xylostella* disappeared. The compromised induced resistance when there are other attackers than *P. xylostella* in the sequence of four attackers, suggests that *B. nigra* plants loose the potential to deal with a specific herbivore attack when switching defence machinery towards other attackers in the sequence. Furthermore, four exposures to attack by the other leaf chewer (*A. rosae*) did not make the plant more resistant to *P. xylostella* caterpillars*.* The absence of induced resistance by the other chewer, indicates that the induced response found on plants attacked four times by *P. xylostella* is not just a general defence mechanism in response to chewers, but that there is specificity in induced defence within feeding guilds (Mertens et al. 2021b). Similar specificity in induced resistance was found for *Solanum dulcamara L.* (bittersweet). Plants that had been damaged by the leaf-chewing beetle *Psylliodes affinis* (Coleoptera, Chrysomelidae) were more resistant to *P. affinis*, while feeding by the leaf chewer *Plagiometriona clavata* (Coleoptera, Chrysomelidae) did not induce resistance against *P. affinis* (Viswanathan et al. 2005). Our work identifies that specificity of induction by herbivore identity may be maintained under multi-herbivore attack.

Plants that were sequentially attacked four times by aphids defended equally well against larvae of *P. xylostella* compared with plants that had not been exposed to herbivory. This indicates that when the plant suffered four rounds of aphid attack there was no aphid-induced susceptibility to a chewer. Several studies of plant responses to dual attack have reported aphid-induced susceptibility to chewers, often supporting their findings based on the negative crosstalk between signal transduction pathways (Davidson-Lowe et al. 2019; Koornneef and Pieterse 2008; Li et al. 2014; Rodriguez-Saona et al. 2005; Soler et al. 2012). The absence of aphid-induced susceptibility to caterpillars or even presence of aphid-induced resistance to caterpillar attack may be caused by aphids depleting nutrients in the plant or by shifts in secondary metabolites (Jakobs et al. 2019). It is becoming clear that not only the guild of previous attackers is important in explaining plant resistance but many other factors such as the density of attackers influence the outcome of plant-mediated interactions between herbivores (Kroes et al. 2015; Pineda et al. 2017). For resistance of *B. nigra* to sequential herbivore attack, the prevalence of the second herbivore in the field is a more important driver of plant-induced responses to the first attacker than the identity of the first herbivore itself (Mertens et al. 2021b).

Our work highlights that also the number of times the plant was attacked by an insect of the same feeding guild within the sequence of four attackers influenced the plant’s capability to respond to attack by *P. xylostella*. Caterpillars grew bigger when feeding on plants that had been exposed to four events of aphid infestation compared with those feeding on plants that had been exposed to four events of chewer attack or compared with plants that had been exposed to two rounds of herbivory by aphids plus two by chewers. This suggests that when the plant suffers attack by herbivores that arrive in sequences, being more times attacked by one type of insect, makes the plant more ready to defend against an insect of a similar type. Moreover, plants attacked by sequences containing two times chewers and two times phloem feeders were more vulnerable to *P. xylostella* larvae when the first attacker was a chewer compared with plants that were first attacked by a phloem feeder. This suggests that the feeding guild of the first attacker may influence the plant’s capability to defend against subsequent attackers. In line with our results, in maize, the order of herbivore arrival was important in determining plant resistance to sequential attack. *Spodoptera frugiperda* attack induced resistance against larvae of *Diabrotica virgifera virgifera*, but only when *S. frugiperda* attacked the plant first (Erb et al. 2011). In our study, the species identity of the first attacker partly influenced plant resistance, as plants that had been attacked first by *M. persicae* were more resistant to *P. xylostella* larvae*.* Moreover, when the last attacker of the sequence was a chewer, especially when it was *A. rosae*, the plant was more resistant to *P. xylostella* larvae*.* Common garden experiments monitoring herbivore communities on *B. nigra* show that *M. persicae* is one of the first attackers colonising *B. nigra* plants, and that *A. rosae* and *P. xylostella* arrive later in the growing season of the plant (Mertens et al. 2021b). The fact that we found herbivore-induced resistance in response to exposure to herbivore sequences that are more commonly found in the field suggests that *B. nigra* plants are adapted to the natural order of herbivore arrival (Mertens et al. 2021b).

Several studies on plant responses against single herbivore attack show canalization of plant responses, where plants attacked by a herbivore cannot fully defend after sequential attack (Soler et al. 2012; Viswanathan et al. 2007). Canalization may not be the optimal defence strategy in a scenario of multiple attack by herbivores that arrive sequentially. If the plant completely directs its defence machinery towards the first herbivore and cannot switch response to the upcoming attackers, the plant may be undefended against later arriving herbivores. Our work does not show evidence for canalization of plant defences in *B. nigra*, as there is no induced susceptibility to *P. xylostella* larvae by sequences of four herbivores, compared to resistance of plants that only received *P. xylostella*. Consequently, when facing herbivory by multiple insects that arrive in sequences, the ability of *B. nigra* plants to defend against a late-arriving herbivore is not hampered. Additionally, our data suggest that *B. nigra* plants do not fully switch their resistance phenotype to a new attacker, i.e., plants that had been attacked four times by *P. xylostella* were more resistant to larvae of *P. xylostella*, but when there was a switch of attackers in between, the induced resistance was reduced. Furthermore, *P. xylostella* larvae grew better on plants attacked by the sequence aphid–chewer–aphid–chewer than on plants attacked by the sequence aphid–aphid–chewer–chewer. The reduced resistance of plants that had been exposed to more switches of attackers further supports that *B. nigra* plants are limited in showing a full defence response when the attacker changes several times.

To conclude, we show that history of sequential attack is an important factor determining plant resistance to its community of attackers. The relative importance of overlapping herbivore populations, densities, timing of their arrival and plant ontogeny, in addition to patterns in incidence we tested here, should be evaluated in future studies. Due to the large number of treatments tested in our study, we could not evaluate how sequential attack by multiple herbivores affects other herbivore species within the same or different feeding guild or level of food plant specialisation. We call for multi-herbivore attack studies to start collecting broader evidence on how different herbivore species perform on multi-herbivore-induced plants and how different plant species cope with similar sequences of herbivore attack. Moreover, in depth studies on the physiological changes after exposure to each newly arriving herbivore should shed light on how plants regulate plasticity to multi-herbivore attack. Additionally, it is crucial that further studies explore plant adaptation to multi-herbivore attack under field conditions. This could be done by studying the herbivore communities forming on plants previously induced by different sequences of herbivory (Stam et al. 2018), assessing the importance of the first attacker, the last attacker and the order of attackers within the sequence and assessing the consequences for other community members and for plant fitness.

## Data Availability

Data will be available upon reasonable request to the corresponding author.
